# Opportunities and obstacles in linking large health care registries: the primary secondary cancer care registry - breast cancer

**DOI:** 10.1186/s12874-022-01601-0

**Published:** 2022-04-27

**Authors:** Marianne J. Heins, Kelly M. de Ligt, Janneke Verloop, Sabine Siesling, Joke C. Korevaar

**Affiliations:** 1 Nivel, Netherlands Institute of Health Services Research, P.O Box 1568, 3500 BN, Utrecht, Netherlands; 2 Department of Research and Development, Netherlands Comprehensive Cancer Organisation, Utrecht, The Netherlands; 3 Department of Health Technology and Services Research, Technical Medical Centre, University of Twente, Enschede, The Netherlands

**Keywords:** Breast neoplasms, Primary care, Registries, Linkage, Big data, Cancer registry, GP registry

## Abstract

**Background:**

The growing volume of health data provides new opportunities for medical research. By using existing registries, large populations can be studied over a long period of time. Patient-level linkage of registries leads to even more detailed and extended information per patient, but brings challenges regarding responsibilities, privacy and security, and quality of data linkage. In this paper we describe how we dealt with these challenges when creating the Primary Secondary Cancer Care Registry (PSCCR)- Breast Cancer.

**Methods:**

The PSCCR – Breast Cancer was created by linking two existing registries containing data on 1) diagnosis, tumour and treatment characteristics of all Dutch breast cancer patients (NCR), and 2) consultations and diagnoses from primary care electronic health records of about 10% of Dutch GP practices (Nivel-PCD). The existing registry governance structures and privacy regulations were incorporated in those of the new registry. Privacy and security risks were reassessed. Data were restricted to females and linked using postal code and date of birth. The breast cancer diagnosis was verified in both registries and for a subsample of 44 patients with the GP as well.

**Results:**

A collaboration agreement was signed in which the organisations retained data responsibility and accountability for ‘their’ registry. A Trusted Third Party performed the record linkage. Ten percent of the patients with breast cancer could be linked to the primary care registry, as was expected based on the coverage of Nivel-PCD, and finally 7 % could be included. The breast cancer diagnosis was verified by the GP in 42 of the 44 patients.

**Conclusions:**

We developed and validated a procedure for patient-level linkage of health data registries without a unique identifier, while preserving the integrity and privacy of the original registries. The method described may help researchers wishing to link existing health data registries.

## Background

Due to rapid digitalization, the volume and availability of health data is expanding fast [1]. The availability of these health data will provide new opportunities for medical research. For example, traditional methods for data collection (e.g. randomised controlled trials, cohort studies and surveys) are often expensive, time intensive and can suffer from low response rates [2]. By linking existing registries, time and budget can be saved as recruitment of new patients and collection of new data is not necessary [2, 3]. Besides, as these registries often include unselected populations and not only those who are eligible and agree to participate in a clinical trial, there is less selection bias and populations that are often under-represented in surveys or clinical trials (such as migrants or elderly) can be studied effectively [2, 3]. Furthermore, the volume and length of follow up in these registries may also enable studying patients in subgroups, different stages, with rare (subtypes of) diseases or rare events and for a longer time [2, 3].

Linkage of existing registries can further increase the potential of health data [2, 4, 5]. Most registries are designed for a specific goal, and only data related to that goal is gathered. For example, clinical registries may contain detailed disease-specific information, but lack detailed information about other medical diagnoses or health care utilization. Administrative registries may contain data on health care utilization, but often lack detailed disease-specific information [6]. Patient-level linkage of registries leads to more detailed and extended information per patient, [3] combining the strengths of the original registries and allowing to study a wider range of research questions.

Besides opportunities, linkage of registries also brings challenges. Most health data are spread across organizations, various servers and networks, and data are sometimes purposely separated to prevent traceability to individuals [1, 7, 8]. Linkage of registries therefore brings challenges regarding 1) responsibilities 2) privacy and security and 3) quality of data linkage. These challenges will be discussed in more detail below.

First, when registries from multiple organizations are linked, responsibility and accountability for data need to be arranged. Negotiating the legal, ethical and governance frameworks and requirements may take considerable time and effort, as they often have to be in line with existing structures [3]. Besides, linkage often requires approval from several institutional bodies, who may all have their own requirements and perspectives [2].

Second, patient privacy and data security are of utmost importance when handling health data, especially when data from different sources are linked; enrichment of registries with more details about a unique person increases the risk that a person can be identified. Appropriate measures should be taken to guarantee privacy protection [5, 8, 9]. Besides, obtaining individual informed consent to link data may not be feasible for large datasets [8].

Third, linkage of patient records must be correct when two registries are combined. Ideally, every person is recognized by a unique identifier that can be used to link registries [2, 5]. In most cases, however, a unique identifier is absent and linkage is based on a combination of several variables that are not absolutely unique, such as name, sex, date of birth, or may differ over time, such as postal code. These linkage variables should be chosen sensibly, and validity and accuracy of linkage should be ascertained [2].

In this paper we will describe how we dealt with the challenges related to responsibilities, security and linkage validity when creating a large registry, the Primary and Secondary Cancer Care Registry (PSCCR) – Breast Cancer. This registry was created from two existing registries, a cancer registry with data on diagnosis, tumour, treatment and a primary care registry with data on primary care contacts and diagnoses.

## Methods

### Data sources

To develop the Primary and Secondary Cancer Care registry (PSCCR) – Breast Cancer we linked data from the NCR and Nivel-PCD, two existing registries that we will first describe in more detail:

#### Netherlands cancer registry (NCR)

The NCR is hosted by the Netherlands Comprehensive Cancer Organisation (IKNL). It contains nationwide data about the diagnosis and treatment of all cancer patients treated in all Dutch hospitals since 1989 [10]. Based on notification by the national pathology archive (PALGA), the hospital discharge registers and haematology laboratories, details on patient and tumour characteristics, diagnosis and treatment are collected directly from the medical records by trained data managers using national and international standardized coding rules.

#### Nivel primary care database (Nivel-PCD)

The Netherlands Institute for Health Services Research (Nivel) collects longitudinal data that are routinely recorded by general practitioners (GPs), and processes these data into Nivel-Primary Care Database (Nivel-PCD) [11]. Nivel-PCD contains data on the number and type of contacts with the primary care physician, health conditions that are presented during these contacts, prescriptions, results of laboratory tests, measurements and referrals to secondary care. Currently, data are collected from a dynamic cohort of approximately 500 practices (representing about 10% of all Dutch practices) spread throughout the Netherlands. All Dutch inhabitants are registered with a primary care physician [12]. Patients in Nivel-PCD are a representative sample of the Dutch population as to age and sex [11].

### Responsibilities

Both the NCR and Nivel-PCD registries have existing governance structures [10, 11] including bodies that had to approve the linkage of both registries. The legal departments of IKNL and Nivel composed a collaboration agreement in which they described responsibilities. The existing governance structures were incorporated into the new governance structure of PSCCR Breast Cancer. Both bodies approved of the linkage of their registries. For future updates with other tumour types, new approval must be asked. However, a simple addendum to the collaboration agreement is sufficient.

### Privacy and security

Both the NCR and Nivel-PCD have their own privacy boards and security measures that remain in force. However, additional privacy measures were necessary to ensure patient privacy and protect the integrity of both individual registries after linkage. Therefore, procedures were developed in which linkage and analysis processes were strictly separated, i.e. they were performed by separate departments and data were stored separately. Clinical data from both registries was only shared once the linkage process was validated and data was shared only for those patients that could be linked.

The privacy protocol, describing these procedures, was assessed by the Privacy review boards of NCR and Nivel-PCD. In addition, a data protection impact analysis (DPIA) was performed by researchers and data analysts of IKNL and Nivel. This is a legally required procedure (art. 35 General Data Protection Regulation) to assess privacy and security risks before handling data, in order to be able to take appropriate measures to reduce any risks. Although the specific procedure may differ, it includes a systematic description of the way data are handled, assessment of privacy and security risks and measures to reduce these risks.

Neither obtaining informed consent from patients nor approval by a medical ethics committee is obligatory for this type of observational studies containing no directly identifiable data (art. 9.2 sub j General Data Protection Regulation, art. 24 Dutch GDPR Implementation Act jo). Patients were not informed explicitly that their data was included in the PSCCR-Breast Cancer. IKNL provides brochures to all hospitals, that can be used to inform patients who are diagnosed with cancer that their data is included in the NCR. All practices participating in Nivel-PCD inform their patients, e.g. via a poster or tv-screen in the waiting room, that their data is included in Nivel-PCD. Both registries provide patients the option to opt-out and have their data removed.

### Quality of data linkage

In the absence of a unique identifier, the two registries were restricted to females and linked using the combination of date of birth and postal code (neighbourhood level, on average 2.000 households [13]). The combination of date of birth and postal code is not unique, as women born on the same day and living in the same neighbourhood will have the same combination.

Women were only included into PSCCR – Breast Cancer when a breast cancer diagnosis was registered in both NCR and Nivel-PCD (ICD-10 code C50 in the NCR and ICPC code X76 in Nivel PCD). For a small random sample of women, the diagnosis was also validated by their primary care provider by providing birth year and month, date of registry with the GP practice, year of diagnosis and date of the last encounter with the primary care provider.

The linkage procedure was tested and the percentage of patients that could be linked was assessed. As Nivel-PCD covers about 10% of the Dutch population, we expected that also 10% of the breast cancer patients identified in NCR could be linked to Nivel-PCD. To assess possible inclusion bias, the percentage of patients that could be linked successfully was compared for several subgroups of patients.

## Results

### Responsibilities

In the collaboration agreement, the goal and methods of the linkage, privacy procedures and mutual responsibilities were described. IKNL and Nivel both declared that they retain the full responsibility and accountability for the data from their own registry and share responsibility and accountability for the data concerning the linkage between both registries. The collaboration agreement was designed in such a way that it allows extension of the existing registry without having to negotiate the legal, ethical and governance frameworks and requirements again. Third parties can request data from PSCCR Breast Cancer when use of these data is in line with the goals of both registries. Linkage to their own data is also possible, but requires a DPIA, approval of the Privacy review boards of NCR and Nivel-PCD, and testing of the linkage procedure. A data dictionary of the PSCCR if available on request from the authors.

### Privacy and security

The privacy protocol was approved by the Privacy review boards of NCR and Nivel-PCD. The DPIA was performed by IKNL and Nivel researchers and data protection officers. The main risks identified with the DPIA were: 1) sensitive data about a large number of patients are included in the PSCCR-Breast cancer, so very strict privacy measures and an opt out procedure were installed 2) several organisations are involved in data handling, making a clear security policy with agreements about responsibilities for data security and handling and reporting of security incidents pivotal.

**Fig. 1:**
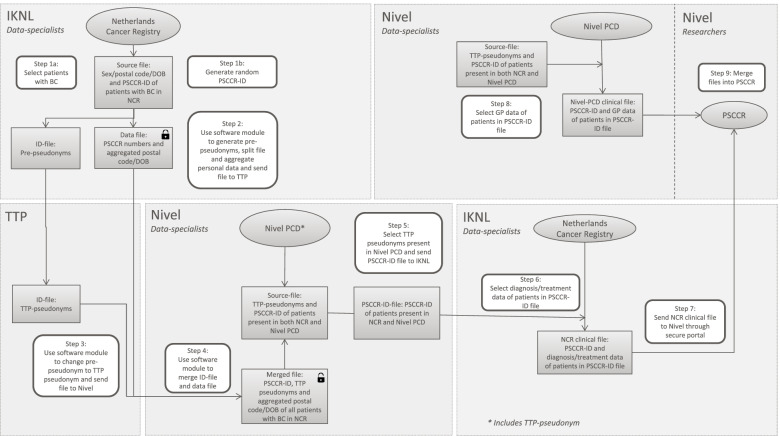
Title: Linkage procedure. DOB = date of birth, Nivel PCD=Nivel Primary Care Database, PC = postal code, PSCCR = Primary Secondary Cancer Care Registry, TTP = trusted third party. Ovals represent the original database, rectangles represent data files that are sent between the parties, rounded rectangles represent steps of the process

Linkage and analysis processes were strictly separated by use of a trusted third party (TTP) (Fig. 1). Before sending the file with personal data to the TTP, the sender (in this case IKNL) used a software module that 1) performed several checks on the file, e.g. a date should be in a pre-specified format 2) transferred the personal data into a so-called pre-pseudonym, a combination of letters and numbers 3) aggregated the personal data, i.e. abbreviated the postal code and only kept the year and quarter of birth. The pre-pseudonyms and aggregated personal data were then sent to the TTP. The TTP used a second software module to transfer the pre-pseudonyms to final pseudonyms. This application did not have access to the aggregated personal data. The receiving party (in this case Nivel) used a third software module to combine the final pseudonym and the aggregated personal data. The researchers analysing the linked data only had access to the de-identified clinical data of women who were present in both registries.

### Quality of data linkage

In the NCR, 214,596 women with a diagnosis of breast cancer between 2000 and 2016 were identified. In 2,040 of these women (1%) the combination of postal code and date of birth was not unique within the NCR. These women were excluded as we could not ascertain correct linkage to Nivel-PCD. Of the remaining 212,556 women, 20,449 (9.6%) were present in Nivel-PCD. This is in line with our expectations, as about 10% of the general Dutch population is present in Nivel-PCD [11].

**Fig. 2:**
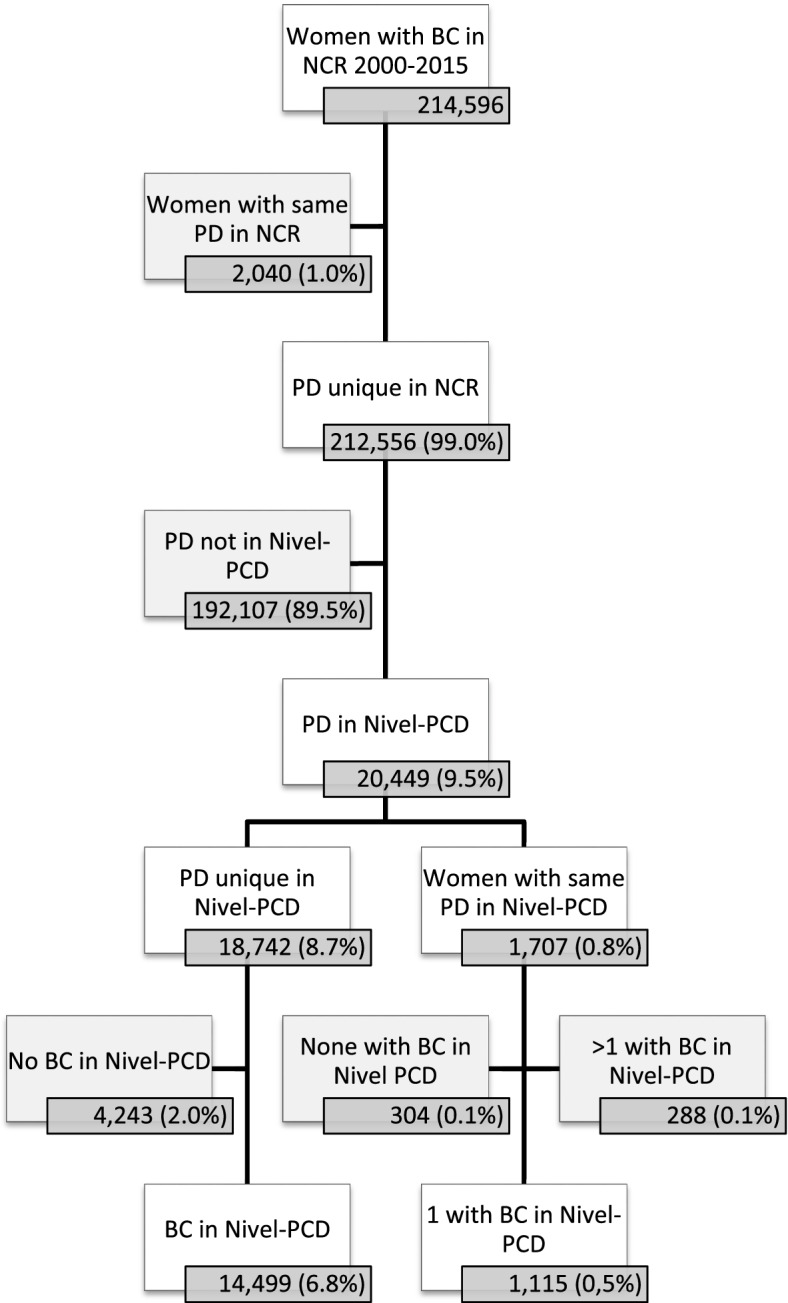
Results of linkage between NKR – NZR. * BC=Breast Cancer, Nivel PCD=Nivel Primary Care Database, NCR = Netherlands Cancer Registry, PD = postal code (4-digits), date of birth. Percentages are calculated according to the total population of women with breast cancer in the NCR

For 18,724 of the 20,499 women, the postal code and date of birth was unique within Nivel-PCD. For 14,499 of them (77%), a diagnosis of breast cancer was also registered in Nivel-PCD, and they were included in PSCCR-Breast Cancer. Another 1,155 women, for whom the postal code and date of birth was not unique within Nivel-PCD, could also be included in PSCCR-Breast Cancer as they were the only woman with that postal code and date of birth who had a diagnosis of breast cancer in Nivel-PCD. So finally, 15,614 women (7%) were included in PSCCR-Breast Cancer (Fig. 2).

To examine the validity of the linkage we compared the percentage of women who were included in the PSCCR – Breast Cancer as to year of diagnosis, age and tumour stage. Women who had been diagnosed a long time ago, older women, and women with advanced disease at diagnosis were less likely to be included (Table 1).

**Table 1:** Characteristics of women included in PSCCR-Breast Cancer and all women diagnosed with breast cancer between 2000 and 2016

Year of diagnosis	All women diagnosed between 2000 and 2016 (*N* = 214,580^a^)		women in PSCCR Breast Cancer (*N* = 15.593)	*p*-value
	N	N	% included	
2000–2004	61,391	3191	5.2%	< 0.001
2005–2009	66,130	4979	7.5%	
2010–2015	87,059	7423	8.5%	
Age at diagnosis				
18–44	25,315	1933	7.6%	< 0.001
45–59	76,198	6100	8.0%	
66–74	73,229	5467	7.5%	
75 and older	39,838	2093	5.3%	
Tumour stage				
DCIS	1481	132	8.9%	< 0.001
I	89,662	7012	7.8%	
II	85,070	6168	7.3%	
III	26,050	1717	6.6%	
IV	10,505	503	4.8%	
Unknown	1812	61	3.4%	
Type of surgery				
Breast conserving	104,314	8318	8.0%	< 0.001
Amputation	89,079	6328	7.1%	
Unknown/Other	511	30	5.9%	
No surgery	20,676	917	4.4%	
Axillary dissection	82,128	5709	7.0%	
(Neo) adjuvant therapy				
Radiotherapy	128,580	10,085	7.8%	
Chemotherapy	80,117	6297	7.9%	
Hormone therapy	110,871	8311	7.5%	

^a^ This number differs slightly from Fig. 2 as these characteristics were not available in the NCR for a small number of women

To check accuracy of linkage, we asked GPs to confirm the breast cancer diagnosis for a small sample of patients included in the PSCCR. Beforehand, we deemed 40 to 50 patients feasible and enough to get a first impression of the accuracy of linkage. We randomly selected practices with a relatively large number of patients and contacted them, until we had validated the diagnosis for 44 patients. Practices were provided with a file with details of their patients from our database that are available at Nivel: year and quarter of birth, date of registration with the practice, year of diagnosis breast cancer in NCR and Nivel PCD, last known GP visit (date and diagnoses made). Based on these variables, the GP could find the patient in their EMR. The GP confirmed the diagnosis in almost all women (*n* = 42). For two women, the GP could not confirm the diagnosis, meaning that they could not find the diagnosis in the medical file or linkage was incorrect.

## Discussion

In this paper we described the opportunities and challenges in linking two large healthcare registries into the PSCCR-Breast Cancer. As to responsibilities, a strength of our method is that the existing governance structures provided the foundation for the governance of the new registry. However, it still turned out to be a rather time-consuming process as the legal departments of both organisations had to review the details of the agreements. Besides, we aimed to create a governance document that would not only cover the current project, but also possible future projects and extensions of the registry with other cancer types or new data.

As to data privacy and security, when using health data for research there is always a dual aim of allowing health data to be used for research that improves public health whilst maintaining public trust that data is used securely and robustly. We applied measures, described in the governance documents, to strictly separate linkage from analysis processes, which is considered as best practice [2]. To objectively weigh the benefit of our database and the research that could be done with it against the risks related to security and privacy, our method was assessed by the privacy committees of IKNL and Nivel. They both concluded that the benefits outweighs the risks. Our method also provides the opportunity to link the PSCCR to other registries in the future. This may bring along new privacy risks, which will have to be assessed by the Privacy review boards of NCR and Nivel-PCD. This also holds for third parties who want to use PSCCR data or link it to their own data.

As to accuracy and validity of data linkage, a strength of our method is that the linkage method can be used to update the database relatively easily and can also be modified for other cancer types. A weakness is the absence of a unique patient identifier in both databases. Our linkage method could have resulted in false linkages. To increase the probability of correct linkage, we required a breast cancer diagnosis in both databases. This increased internal validity of the database, but it is a strong requirement and we may have missed women for whom the diagnosis of breast cancer was not registered by the GP. The diagnosis data did not have to agree within a specified time period, as that would have made the requirement even stronger. Patients who moved to another neighbourhood after the breast cancer diagnosis will not have been included in the PSCCR, as they could not be matched, and might even incorrectly have been included as controls. This will be more common in women diagnosed a longer time ago and may dilute effects of case-control studies performed with PSCCR data. We expect that with a unique patient identifier, a higher number of patients could be linked with a higher accuracy.

The PSCCR – Breast Cancer has several strengths. First, the registry includes a large unselected population, so elderly patients or patients receiving specific treatment combinations can be studied. Second, the follow-up period of patients more than 15 years, which allows studying patients from the pre-diagnostic phase up to many years after diagnosis. Third, an unselected reference group of women without breast cancer can be retrieved from Nivel-PCD, so comparisons can be made with age-matched controls [14]. Internal validity of the underlying registries is good, as they have strict quality checks and researchers that are highly familiar with the registry. External validity is also expected to be high, as both registries contain an unselected population.

The use of these existing databases with observational data also has weaknesses. Data were not specifically collected for research purposes, but registered for patient care, and may therefore be incomplete. Some subgroups turned out to be somewhat underrepresented in the PSCCR – Breast Cancer, i.e. patients who died relatively shortly after the diagnosis or patients who were diagnosed a longer time ago. This is because they were less likely to be registered in one of the Nivel-PCD practices during the period of data collection for the PSCCR. As these groups are often entirely excluded from clinical trials, their inclusion in our database is still an advantage. However, this means that our database cannot be used to determine the prevalence or incidence of breast cancer, or give a description of the entire population of women who have been diagnosed with breast cancer.

## Conclusions

We developed and validated a method for patient-level linkage of health data registries without a unique identifier, while preserving the integrity, privacy and responsibility for the original registries. The PSCCR-Breast Cancer registry that we created combines the strength of two existing registries in a way that their data enrich each other and allows studying a wide range of research questions related to breast cancer, from the diagnostic pathway to adverse effects of treatments and palliative care. The opportunities and obstacles described may form a blueprint for linking existing healthcare registries.

## Data Availability

The datasets used and/or analysed during the current study are available from the corresponding author on reasonable request.

## Abbreviations

PSSCR: Primary and Secondary Cancer Care Registry
NCR: Netherlands cancer registry
Nivel-PCD: Nivel Primary Care Database
GP: General practitioner
DPIA: Data protection impact analysis
TTP: Trusted Third Party
